# Diabetes prevention among American Indians: the role of self-efficacy, risk perception, numeracy and cultural identity

**DOI:** 10.1186/s12889-017-4766-x

**Published:** 2017-10-02

**Authors:** Vanessa W. Simonds, Adam Omidpanah, Dedra Buchwald

**Affiliations:** 1 Department of Health and Human Development, Montana State University, Bozeman, MT USA; 2 College of Nursing, Initiative for Research and Education to Advance Community Health (IREACH), Washington State University, Spokane, WA USA; 3 Elson S. Floyd College of Medicine, Initiative for Research and Education to Advance Community Health (IREACH), Washington State University, Spokane, WA USA

**Keywords:** American Indians, Risk perception, Diabetes, Self-efficacy, Numeracy

## Abstract

**Background:**

According to the Risk Perception Attitude (RPA) framework, classifying people according to their perceptions of disease risk and their self-efficacy beliefs allows us to predict their likelihood for engaging in preventive behaviors. Health interventions can then be targeted according to RPA group. We applied the framework to type 2 diabetes prevention behaviors among American Indians and expanded it to include culture and numeracy.

**Methods:**

Using a cross-sectional study design, we surveyed a sample of Northern Plains American Indians in a reservation community setting on self-reported perceptions of diabetes risk, objective diabetes risk, self-efficacy, engagement in healthy behaviors, knowledge of diabetes risk factors, and covariates including demographics, numeracy, and cultural identity. We used the RPA framework to classify participants into four groups based on their perceptions of risk and self-efficacy. Analyses of variance and covariance estimated inter-group differences in behaviors associated with type 2 diabetes prevention.

**Results:**

Among 128 participants, our only finding consistent with the RPA framework was that self-efficacy and risk perception predicted knowledge about diabetes risk factors. We found limited evidence for the influence of cultural identity within the RPA framework. Overall, participants had lower numeracy skills which tended to be associated with inaccurate perceptions of higher levels of risk.

**Conclusions:**

The theoretical framework may benefit from inclusion of further contextual factors that influence these behaviors. Attention to numeracy skills stands out in our study as an important influence on the RPA framework, highlighting the importance of attending to numeracy when targeting and tailoring risk information to participants segmented by the RPA framework.

## Background

Type 2 diabetes (hereafter “diabetes”) is a leading cause of death and disability for American Indians (AIs) [1]. According to national data for 2010–2012, diabetes was twice as likely to affect AI men and women as compared to white men and women [2, 3]. Higher rates of obesity also disproportionately affect both AI men compared to white men (33.9% vs 23.3%) and AI women compared to white women (35.5% vs. 21.0%) [2]. These disparities underscore the need for well-informed interventions designed for AIs.

Improving diet and increasing physical exercise are widely endorsed approaches for reducing diabetes risk [4–8]. Many health behavior models propose that risk perception is a key contributor to people’s willingness to undertake these behavioral changes [9–11]. However, the evidence for this proposition is ambiguous since some studies have found a link between perceived risk and adoption of healthy behaviors [12, 13], while others have not [14, 15]. One reason for these conflicting results is explained by the Risk Perception Attitude (RPA) framework [16], which hypothesizes that individual self-efficacy beliefs modify the behavioral effects of risk perception. Substantial research has found that self-efficacy modifies the effect of risk perception on health behaviors related to the prevention of HIV [17], HPV [18], cancer [19–21], diabetes [19, 22], as well as behaviors related to nutrition [23], and smoking [24].

**Fig. 1:**
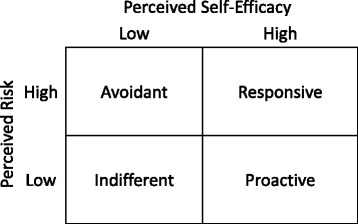
Risk Perception Attitude Framework Categories

The objective of the RPA framework is to target interventions and educational materials based on the type of individual as classified by one of four RPA groups: “Avoidant” individuals with high perceived risk and low self-efficacy. “Indifferent” individuals with low perceived risk and low self-efficacy. “Proactive” individuals with low perceived risk offset by high self-efficacy. “Responsive” individuals with heightened perceived risk and self-efficacy (see Fig. 1).

From a theoretical standpoint, understanding the determinants of risk perception can inform more effective interventions to promote behaviors that reduce risk. An important characteristic neglected by the RPA framework is the influence of low numeracy skills on people’s ability to understand and information about their their risk for specific illnesses [25, 26]. Several studies indicate that numeracy skills affect interpretations of risk [27, 28]. In addition, limited numeracy can also inhibit people’s ability to take health-related actions on the basis of quantitative information [25]. For example, understanding numeric nutrition information, such as caloric intake and appropriate serving sizes, can be a burden for those with low numeracy skills, in turn, leading to higher BMI [29]. Numeracy was associated with greater knowledge and healthier diabetes related behaviors in a large sample of AIs with diabetes. [30, 31] Yet, despite the importance of numeracy in risk perception and health behaviors, no previous studies have considered its potential role in the RPA framework. Since very little is known about numeracy and its role in accurately perceiving diabetes risk, we propose exploratory analyses to examine its role in the RPA framework.

Although considerable research has addressed diabetes among AIs, few studies have explored AI perceptions of diabetes risk or the association of these perceptions with personal and demographic factors, including cultural identity [22, 32]. The only study using the RPA framework with AIs proposed that the framework could be enhanced by attending to cultural characteristics that may influence both perceptions of risk, self-efficacy, and behavioral intention. [22] Values and traditions are shaped by cultural identity. In the case of diabetes, cultural identity might influence awareness of risk by affecting a person’s understanding of what counts as a healthy diet or a healthy body [33, 34].

The RPA framework has been used to predict knowledge and information seeking, as well as behavioral outcomes [17, 19, 20, 22, 23, 35]. The primary focus of this paper is to apply the framework in the context of prevention of diabetes among AIs. First, we examine whether we can predict diabetes knowledge, which includes awareness of its primary risk factors; diet; exercise; and genetics. Second, we examine whether it can predict active engagement in behaviors that prevent diabetes which include those related to diet and exercise. Both the disposition to adopt a particular behavior and active engagement in that behavior can be measured with reference to five stages of change: precontemplation, contemplation, preparation, action, and maintenance. In the first stage, people do not even consider engaging in the behavior, while in the fifth stage, they regularly practice it [36, 37].

The limited available evidence suggests that risk perception has a complex relationship with health behaviors for AIs [22, 32]. This study is innovative as the first study to explore the impact of risk perception and self-efficacy on diabetes prevention related knowledge and behavior in AI communities. The objective of the present study was to answer the question of whether assignment to one of the four RPA categories, defined by one’s self-efficacy and risk perception, can predict knowledge about diabetes risk factors and stage of change for engaging in diabetes preventive behaviors. Furthermore, we advanced the RPA theory by examining the potential contributions of numeracy and cultural characteristics in predicting risk perception both of which may be important contributors to accurate predictions using the RPA framework. We hypothesized that we could use self-efficacy and perception of risk to predict knowledge about diabetes risk factors and stage change for diabetes preventing behaviors of diet and exercise. We predict that together, higher perception of diabetes risk and higher self-efficacy, increase the stage of change a person is in and are associated with higher levels of knowledge.

## Methods

### Data collection

This cross-sectional study included a convenience sample of 143 men and women all self-identified members of the same Northern Plains Tribe recruited at two separate community events on a Northern Plains reservation. The first event was a local craft fair in December 2013; the second was a powwow in September 2014. Attendees were invited to take the survey as they walked by the researchers’ booth. Many survey respondents recruited other tribal members to take the survey. After demonstrating eligibility (being a local tribal member over 18 years of age without diagnosed diabetes) and providing informed consent, participants completed the survey, which required about 15–25 min. This study was approved by local tribal council and approved by Montana State University IRB.

### Measures

Participants filled out a self-administered, 47-item survey to measure the main constructs of the RPA framework: perceptions of diabetes risk and self-efficacy. The survey also included items related to the outcomes we intended to predict, diabetes knowledge and readiness to engage in diabetes preventing behaviors, along with selected covariates (demographics, numeracy skills, cultural identity, and objective risk of diabetes).

#### Demographics

Demographic data included age, sex, marital status, and educational attainment. Education was coded into less than high school, high school, or college or vocational degree. Marital status was coded as married versus not.

#### Diabetes risk perception

We measured diabetes risk perception using two previously validated items [38] scored with a visual analog scale ranging from 0 to 100%: “What do you think your risk or chance is for developing diabetes in your lifetime?” and “What do you think your risk or chance is for developing diabetes in the next 10 years?”

#### Self-efficacy

We included self-efficacy measures specific to diet and exercise [39]. The diet measure includes five barriers to healthful eating, such as having to rethink their entire way of nutrition, not having support from others and having to make a detailed plan. The exercise measure includes five barriers to carrying out their intentions to exercise, such as feeling depressed, tense, worried, tired or busy. Each item has four possible responses, ranging from 1 for "very uncertain" to 4 for "very certain." Summing responses to the five items created separate self-efficacy scores for diet and exercise.

#### Formation of four risk perception attitude groups

We used perceived risk and self-efficacy scores to classify respondents into four RPA groups, separately for diet and for exercise. The four RPA groups are classified as follows: “Avoidant” (high risk, low self-efficacy), “Indifferent” (low risk, low self-efficacy), “Proactive” (low risk, low self-efficacy), and “Responsive” (high risk, high self-efficacy). We used the approach of Rimal et al. [17], to segment the sample into RPA groups. Sum scores for perceived risk and diet and exercise self-efficacy had very good internal consistency (Cronbach alpha: 0.81, 0.94, 0.95 respectively, mean (SD): 36 (27), 3.2 (0.74), 3.1 (0.83)). Perceived risk had low correlation with self-efficacy (diet 0.15, exercise 0.05). Plots of sum-scores did not present 4 clusters, so rather than using clustering methods, we used principal components analyses to divide participants into RPA groups. Again, separate analyses were undertaken for diet and exercise. Self-efficacy and perceived risk were combined in a dataset and dimensionality was assessed using a scree plot. The two leading eigenvalues were 4.1 and 1.7 followed by values less than 0.4 which we took to be evidence of a two-factor model corresponding to perceived risk and self-efficacy, as expected. The two leading principal components formed a biplot with quadrants representing each risk classification. Participants in the upper right quadrant were classified as responsive (see fig. 1), and likewise for other RPA classifications based on their location. Separate classifications were created based on self-efficacy for diet and exercise.

#### Stages of change

We used validated stages of change measures to assess the adoption of, or intentions to adopt, diet and exercise behaviors [36, 37]. Stage of change for exercise was measured with a single item on regular exercise, defined as planned exercise intended to increase fitness and performed 3–5 times per week for 20–60 min per session. Participants chose one of five statements to indicate their readiness to undertake regular exercise. Stage of change for diet was measured with eight items on dietary constituents, including low-fat foods as well as fruits and vegetables. Responses were categorized into the five stages noted in the introduction: precontemplation, contemplation, preparation, action, and maintenance.

#### Knowledge of diabetes risk factors

We used the knowledge scale developed and used in conjunction with the Risk Perception Survey for Developing Diabetes (RPS-DD) [40, 41]. Respondents rate whether each statement influences the risk of someone getting diabetes. The number of correct responses is summed to give a score ranging from 0 to 11 [40].

#### Numeracy

We used validated items to assess the following numeracy skills: interpreting probability, converting a percentage to a proportion, and converting a proportion to a percentage. Numeracy scores were based on the total number of correct responses 0 to 3 [27, 42].

#### Cultural identity

We assessed cultural characteristics using two separate and independent validated scales [43]. One scale used three questions to define AI cultural identity. The other scale used two questions to measure white cultural identity. Each question on both scales was scored 1 for not at all, 2 for a little, 3 for some, and 4 for a lot. Mean scores were calculated for the three AI cultural identity items and two white cultural identity items.

#### Objective measure of diabetes risk

We calculated a validated objective risk score [44]. The score ranges from 0 to 10 based on risk factors including age, sex, history of gestational diabetes, family history of diabetes, blood pressure, exercise, and weight. Scores of 5 and above indicate high risk of developing diabetes; scores below 5 indicate low risk.

### Data analyses

We generated descriptive statistics, including means with standard deviations for continuous variables and frequencies for categorical variables. We conducted complete case analyses. Analyses of variance (ANOVA) were used to test for differences in stage of change and knowledge between RPA groups. Analyses of covariance (ANCOVA) were used to test for interaction of effects by numeracy and cultural identity on knowledge as applicable. Where noted, models were expanded to control for the influence of possibly confounding factors: age, sex, education, and marital status. No control was made for BMI as we believe it may collide the effect of action and maintenance. The Spearman correlation was measured to inspect the calibration between perceptions of lifetime and one-year risk (on a 0–100 probability scale) and objective risk score (on a 0–10 non-probabilistic scale). As a post hoc analysis, we considered numeracy and knowledge as predictors of calibration of objective and perceived risk. To measure agreement between self-assessed diabetes risk and objective diabetes risk and to test the secondary hypothesis that participants with a higher level of numeracy had better agreement, we dichotomized the predictor variables, numeracy and knowledge for ease of interpretation. Statistical testing was conducted at a 0.05 level. R version 3.3.3 was used to conduct all analyses [45].

## Results

### Sample characteristics

Among the 143 participants who completed the survey, the mean age was 41.4 years, 86% were female, 88% had at least a high school education, and 73% were overweight or obese. The AI cultural identity mean score (3.3) was substantially higher than the mean score for white cultural identity (2.6). In the assessment of numeracy, only 9.2% of participants answered all three items correctly, and only 29.4% answered at least two items correctly. With regard to diabetes risk factors, among women, 15.7% reported a diagnosis of gestational diabetes. Among all participants, 17.6% reported a diagnosis of high blood pressure, and 51.4% reported a family history of diabetes. Regular exercise was reported by 77.9%.

### Characteristics of risk perception attitude groups

Respondents were categorized into the four RPA groups (“Avoidant,” “Indifferent,” “Proactive,” “Responsive”) using perceived risk and diet self-efficacy. We conducted complete case analyses. Table 1 shows characteristics of each of the four categories of the RPA for diet. Fifteen respondents did not have complete data for perceived risk and/or diet self-efficacy. Respondents were also classified into four RPA groups using perceived risk and exercise self-efficacy. Table 2 shows characteristics of each of these four RPA groups. Sixteen participants did not answer perceived risk and/or exercise self-efficacy survey questions completely. Many participants had the same scores for self-efficacy of diet and therefore were assigned to the same RPA group. Thus, the RPA groups did not have equal number of participants. There were no significant differences in demographics or diabetes risk factors across the four RPA groups in both diet and exercise.

**Table 1:** Results by demographic and health characteristics for each RPA group for diet

	Responsive (*N* = 35)	Proactive (*N* = 33)	Avoidant (*N* = 22)	Indifferent (*N* = 38)
Age; *mean (sd)*	41.6 (17.2)	41.2 (15.1)	36.0 (16.2)	38.7 (11.8)
Female; *n (%)*	29 (82.9)	29 (87.9)	17 (77.3)	35 (92.1)
Marital status; *n (%)*				
Married	14 (41.2)	14 (45.2)	10 (47.6)	19 (51.4)
Divorced	5 (14.7)	5 (16.1)	3 (14.3)	5 (13.5)
Widowed	1 (2.9)	3 (9.7)	0 (0.0)	1 (2.7)
Never married	14 (41.2)	9 (29.0)	8 (38.1)	12 (32.4)
Education; *n (%)*				
Some high school	5 (15.2)	2 (6.7)	2 (9.5)	4 (10.5)
HS grad or GED	8 (24.2)	3 (10.0)	7 (33.3)	4 (10.5)
Some college	14 (42.4)	16 (53.3)	9 (42.9)	20 (52.6)
College graduate	6 (18.2)	9 (30.0)	3 (14.3)	10 (26.3)
BMI; *n (%)*				
Normal	10 (28.6)	8 (25.8)	7 (35.0)	9 (24.3)
Overweight	11 (31.4)	8 (25.8)	8 (40.0)	9 (24.3)
Obese	14 (40.0)	15 (48.4)	5 (25.0)	19 (51.4)
Stage of Change: Physical Exercise; *n (%)*				
Maintenance	10 (32.3)	11 (33.3)	4 (19.0)	10 (26.3)
Action	10 (32.3)	5 (15.2)	9 (42.9)	8 (21.1)
Preparation	6 (19.4)	13 (39.4)	5 (23.8)	12 (31.6)
Contemplation	2 (6.5)	4 (12.1)	3 (14.3)	8 (21.1)
Precontemplation	3 (9.7)	0 (0.0)	0 (0.0)	0 (0.0)
Stage of Change: Eating fruit and vegetables; *n (%)*				
Maintenance	18 (54.5)	19 (57.6)	6 (28.6)	15 (39.5)
Action	8 (24.2)	7 (21.2)	5 (23.8)	13 (34.2)
Preparation	3 (9.1)	4 (12.1)	10 (47.6)	6 (15.8)
Contemplation	4 (12.1)	2 (6.1)	0 (0.0)	3 (7.9)
Precontemplation	0 (0.0)	1 (3.0)	0 (0.0)	1 (2.6)
Gestational diabetes^a^; *n (%)*	2 (6.5)	3 (10.0)	1 (5.3)	12 (33.3)
High blood pressure; *n (%)*	3 (8.6)	8 (24.2)	2 (9.1)	8 (21.1)
Family history diabetes; *n (%)*	11 (31.4)	18 (54.5)	10 (45.5)	26 (68.4)
Numeracy; *mean (sd)*	0.7 (0.8)	1.0 (1.0)	0.8 (1.0)	1.3 (1.0)
American Indian cultural identity; *mean (sd)*	3.4 (0.7)	3.6 (0.6)	3.1 (0.9)	3.4 (0.9)
White cultural identity; *mean (sd)*	2.6 (0.7)	2.7 (0.9)	2.6 (1.0)	2.8 (0.7)
Knowledge; *mean (sd)*	5.0 (3.3)	7.2 (1.7)	5.6 (2.9)	6.9 (2.6)

^a^Among women only

**Table 2:** Results by demographic and health characteristics for each RPA group for exercise^a^

	Responsive (*N* = 30)	Proactive (*N* = 32)	Avoidant (*N* = 39)	Indifferent (*N* = 26)
Age; *mean (sd)*	41.3 (12.9)	39.8 (18.6)	38.4 (12.3)	42.1 (16.4)
Female; *n (%)*	25 (83.3)	26 (81.2)	36 (92.3)	21 (80.8)
Marital status; *n (%)*				
Married	13 (46.4)	12 (38.7)	21 (55.3)	10 (40.0)
Divorced	2 (7.1)	5 (16.1)	5 (13.2)	7 (28.0)
Widowed	2 (7.1)	0 (0.0)	2 (5.3)	1 (4.0)
Never married	11 (39.3)	14 (45.2)	10 (26.3)	7 (28.0)
Education; *n (%)*				
Some high school	3 (11.5)	5 (16.1)	1 (2.6)	3 (12.0)
HS grad or GED	5 (19.2)	8 (25.8)	3 (7.7)	6 (24.0)
Some college	11 (42.3)	14 (45.2)	22 (56.4)	12 (48.0)
College graduate	7 (26.9)	4 (12.9)	13 (33.3)	4 (16.0)
BMI; *n (%)*				
Normal	8 (29.6)	10 (31.2)	7 (18.4)	8 (32.0)
Overweight	5 (18.5)	12 (37.5)	10 (26.3)	8 (32.0)
Obese	14 (51.9)	10 (31.2)	21 (55.3)	9 (36.0)
Stage of Change: Physical Exercise; *n (%)*				
Maintenance	13 (43.3)	6 (21.4)	10 (25.6)	5 (20.0)
Action	7 (23.3)	9 (32.1)	9 (23.1)	7 (28.0)
Preparation	6 (20.0)	8 (28.6)	13 (33.3)	9 (36.0)
Contemplation	4 (13.3)	5 (17.9)	7 (17.9)	1 (4.0)
Precontemplation	0 (0.0)	0 (0.0)	0 (0.0)	3 (12.0)
Stage of Change: Eating fruit and vegetables; *n (%)*				
Maintenance	15 (50.0)	14 (46.7)	18 (46.2)	10 (40.0)
Action	11 (36.7)	7 (23.3)	11 (28.2)	5 (20.0)
Preparation	1 (3.3)	6 (20.0)	7 (17.9)	8 (32.0)
Contemplation	3 (10.0)	3 (10.0)	2 (5.1)	1 (4.0)
Precontemplation	0 (0.0)	0 (0.0)	1 (2.6)	1 (4.0)
Gestational diabetes^b^; *n (%)*	4 (16.0)	1 (3.7)	8 (21.1)	3 (12.5)
High blood pressure; *n (%)*	7 (23.3)	3 (9.4)	8 (20.5)	4 (15.4)
Family history diabetes; *n (%)*	17 (56.7)	11 (34.4)	27 (69.2)	9 (34.6)
Numeracy; *mean (sd)*	1.0 (0.9)	0.7 (0.8)	1.3 (1.1)	0.9 (1.1)
American Indian cultural identity; *mean (sd)*	3.3 (0.8)	3.4 (0.7)	3.4 (0.7)	3.4 (0.9)
White cultural identity; *mean (sd)*	2.6 (0.9)	2.5 (0.7)	3.0 (0.7)	2.7 (0.9)
Knowledge; *mean (sd)*	6.9 (2.5)	5.3 (3.4)	6.8 (2.5)	6.0 (2.8)

^a^One person did not answer all of the self-efficacy questions for exercise

^b^Among women only

### Testing RPA group effects on knowledge and stage of change

**Fig. 2:**
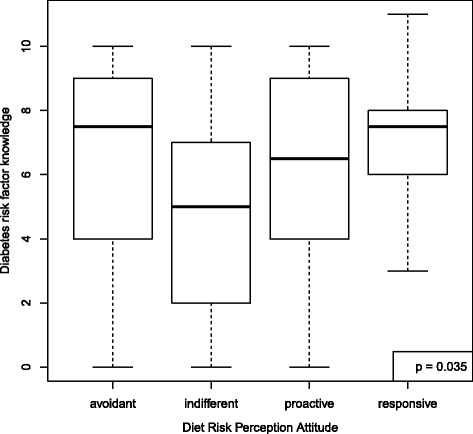
Boxplot of knowledge according to RPA category. RPA groups categorized using self-efficacy for engaging in healthy diet behaviors and perceived risk for developing diabetes. All models adjusting for age, sex, education, and marital status

**Fig. 3:**
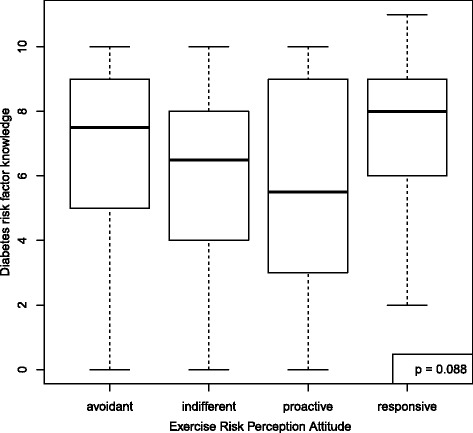
Boxplot of knowledge according to RPA category. RPA groups categorized using self-efficacy for engaging engaging in exercise and perceived risk for developing diabetes. All models adjusting for age, sex, education, and marital status

**Fig. 4:**
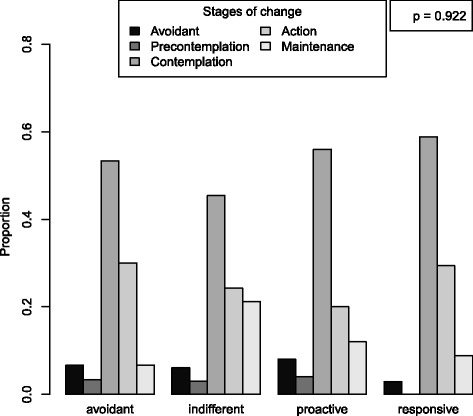
Barplot of Stage of Change for diet according to RPA category. RPA groups categorized using self-efficacy for engaging healthy diet behaviors and perceived risk for developing diabetes. All models adjusting for age, sex, education, and marital status

**Fig. 5:**
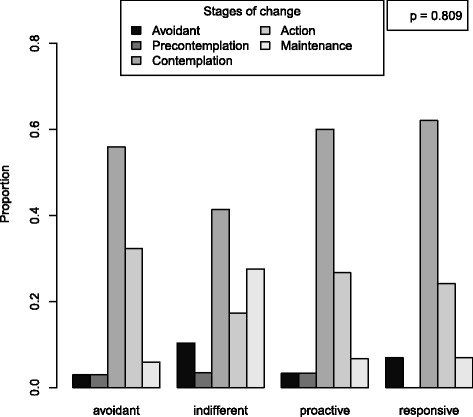
Barplot of Stage of Change for exercise according to RPA category. RPA groups categorized using self-efficacy for engaging in exercise and perceived risk for developing diabetes. All models adjusting for age, sex, education, and marital status

The mean score for the outcome, knowledge of diabetes risk factors, was 6.3 (SD = 2.8) out of 11. For diet but not exercise self-efficacy, knowledge scores differed according to RPA category (*p* = 0.035 diet, *p* = 0.088 exercise) (Figs. 2 and 3). For diet RPA, participants classified as “indifferent” were the least knowledgeable about diabetes risk factors (mean 4.7, SD 3.3). For exercise RPA, “proactive” participants were the least knowledgeable (mean 5.3, SD 3.3). RPA group membership did not predict stage of change for diet nor exercise (*p* = 0.922 for diet and *p* = 0.809 for exercise) (Figs. 4 and 5).

### Expanding RPA framework through further analysis of perceived risk

#### Calibration of perceived risk

Of the 128 participants, 15% of were at high risk for developing diabetes in their lifetime based on the objective risk score. In comparison, the mean score for perceived lifetime risk of diabetes was 34.8 (SD = 26.2), and the mean score for perceived one-year risk was 28.6 (SD = 27.9), both based on a 100-point scale. There were no statistically significant relationships between perceived lifetime risk and objective risk scores. The association between objective and perceived risk had a Spearman correlation of 0.12 (*p* = 0.18). However, the association between objective and perceived risk was more pronounced in participants with more diabetes knowledge (rho = 0.290, *p* = 0.032) and less pronounced in those with less knowledge (rho =0.144, *p* = 0.281). Of the items used to calculate objective risk, the item regarding family history of diabetes had the strongest association with self-reported perceived risk. Participants reporting a family history of diabetes were more likely to have a higher self-reported perceived risk.

#### Numeracy and cultural identity

Regression models showed a bivariate relationship between numeracy and perceived lifetime risk, such that perceived risk was 9.4 points higher for each correct response on the measure (95% CI: 1.8, 10.6). However, controlling for educational attainment attenuated this effect to the point of non-significance (effect 4.4; 95% CI: −0.3, 9.1). The association between objective and perceived risk was more pronounced in participants with high numeracy (rho = 0.281) and less pronounced in those with low (rho =0.168) levels of numeracy, but they were not statistically significant. Numeracy was a significant predictor of diabetes risk factor knowledge such that participants differing by one correct response in our scale had a 0.67 point higher diabetes risk factor knowledge (95% CI: 0.15, 1.19), however numeracy did not modify the association between RPA and knowledge (*p* = 0.351 for diet and *p* = 0.412 for exercise). Adjustment for cultural identity was not significant and did not affect any of the results.

## Discussion

Despite the high rates of diabetes and its complications, the scarcity of information on risk perception and health behaviors is especially notable for AIs. We examined whether risk perception and self-efficacy can predict knowledge and behavioral intention. Understanding the relationship of self-efficacy and risk perception to these targeted outcomes is useful for segmenting audiences and directing health messages appropriately. Although 61.9% of participants in our study perceived that they were at or above a 20% lifetime risk of developing diabetes, perceptions of higher risk did not predict diet or exercise behavioral intentions. Even when we combined self-efficacy with risk perception to assign participants to RPA group, we were unable to predict diet and exercise behavioral intentions. Our only finding consistent with the RPA framework was that diet RPA group predicted knowledge of diabetes risk factors. “Indifferent” participants, meaning those with low perceived risk and low self-efficacy, were the least knowledgeable. This finding confirms previous studies testing the RPA framework to predict knowledge related outcomes [17, 23, 35]. For example, “indifferent” participants in a study of HIV had less knowledge of HIV risk than participants in the other categories [17]. However, in contrast to the RPA framework, we found exercise “proactive” individuals had the lowest knowledge scores compared to other exercise RPA groups.

The inability to accurately predict diet and exercise behaviors may be due to their unique nature. The context regulating diet and exercise is much more complex than knowledge or other behaviors previously targeted by the RPA framework [46–48]. For example, access to healthy food or places to engage in exercise, or the impact of family and friends on behavioral intentions and risk perception may not be reflected in the measures we used. In addition, we measured targeted outcomes of diet and exercise behavioral intentions without specific mention of diabetes prevention. Previous studies using the RPA framework often measure the targeted outcome by explicitly associating it with the health outcome that people are perceiving themselves to be at risk for. For example, one study examined perceived cancer risk and the outcome, cancer screenings [23]. Although our measure of self-efficacy was directly related to the targeted health outcome, our study suggests that accurate behavioral predictions using the RPA framework may also require targeted health outcomes (diabetes) that are explicitly mentioned, as opposed to the general diet and exercise stage of change measures we used. Our use of the RPA framework assumed that participants were aware that diet and exercise influence diabetes prevention, which may or may not be true.

Our study is the first to examine the role of numeracy within the RPA framework, which may be particularly relevant to risk perception [28, 49]. Consistent with recent research with AI adults [27, 42], study participants had low numeracy skills which were associated with inaccurate perceptions of heightened risk [27, 50]. These findings highlight pressing needs for educational materials to assist AI adults with limited numeracy understand their diabetes risk, as well as for community education campaigns to account for and even improve numeracy skills among AIs. Similarly, we found that people with higher diabetes knowledge scores had more accurate perceptions of their own risk for developing diabetes, underscoring the importance of providing appropriate health information to improve accurate perceptions of risk.

We also included an exploratory analysis of the influence of cultural identity on risk perception. Despite widespread agreement that culture is a key factor in perceiving disease risk and understanding disease processes [51, 52] and that our sample identified more closely with American Indian cultural identity, we did not find a relationship between our measure of cultural identity and risk perception. However, while our measure of cultural identity provides more insight than check-the-box measures of racial ancestry, the measure of cultural identity we used does not allow for deeper reflection on the cultural influences on the relationship between risk perception and behavior. A previous study of the RPA framework among AI women found that participants scored high on quantitative measures of self-efficacy but low on qualitative measures, indicating the need for better understanding of cultural meanings related to self-efficacy in AIs [22]. Further in-depth analysis of self-efficacy and its impact on behavioral intention in this population is needed.

This research has certain limitations. First, this population sample was not representative of AIs nationwide. Most participants were older than 40 and although they reported not having diabetes, it is likely our sample included people with undiagnosed diabetes. Most participants (54–73%) indicated that they were in the action or maintenance stages for regular exercise (73%) and healthy diet (54%). These findings conflict with national studies reporting high rates of physical inactivity and low rates of fruit and vegetable consumption among AIs [2]. In addition, previous studies with AI/ANs at risk for diabetes did not have as many participants in the action or maintenance stages for regular exercise (37–39%) or healthy diet (42%) [53, 54]. Another limitation of our study is that the RPA categories depend on sample-specific characteristics. In a relatively homogeneous population such as the one in this study, these categories might not be discriminating enough to identify salient differences between subgroups. Future research should focus on the development of a measure of risk perception that is grounded in classical test theory and has the capacity to compare characteristics that modify health behaviors across racial and ethnic groups.

This research also incorporates notable strengths. It is the first study with a large enough sample to explore the RPA framework with AIs, a population with high prevalence of diabetes and recognized needs for effective interventions. This study used measures of self-efficacy specific to diet and exercise and applied stages of change to assess these behaviors. The approach of this study improves on previous studies that assessed self-efficacy for preventing disease in general, rather than focusing on specific conditions and specific behaviors to reduce risk.

## Conclusions

The results of this study provide limited support for using the RPA framework to segment audiences for the purpose of tailoring and targeting health messaging on improving diet and exercise behaviors without explicitly linking them to diabetes prevention. Future research should examine the contextual factors that influence diet and exercise in conjunction with risk perception and self-efficacy. The key findings from our exploratory study were that perceptions of diabetes risk were often inaccurate, and numeracy and knowledge played a critical role in understanding risk. Accordingly, educational materials that present numeric information in an accessible, easily understandable format may be most effective. In addition, educating people about diabetes risk factors may promote more accurate perceptions of risk. This study also identified the need for a theoretical framework that can aid researchers and educators in understanding how perceptions of diabetes risk affect engagement in healthy diet and exercise behaviors among AIs. We suggest inclusion of contextual factors which may include more nuanced measures of culture, as well as greater attention to culture’s influence on both self-efficacy and risk perception.

## Abbreviations

AI: American Indians
RPA: Risk Perception Attitude
